# Design of a peptide-based vaccine against human respiratory syncytial virus using a reverse vaccinology approach: evaluation of immunogenicity, antigenicity, allergenicity, and toxicity

**DOI:** 10.3389/fimmu.2025.1546254

**Published:** 2025-03-28

**Authors:** Hadeel Alnajran, Maaweya Awadalla, Fahad M. Aldakheel, Intikhab Alam, Afaque A. Momin, Wael Alturaiki, Bandar Alosaimi

**Affiliations:** ^1^ Department of Clinical Laboratory Sciences, College of Applied Medical Sciences, King Saud University, Riyadh, Saudi Arabia; ^2^ Research Center, King Fahad Medical City, Riyadh Second Health Cluster, Riyadh, Saudi Arabia; ^3^ Center of Excellence for Smart Health (KCSH), King Abdullah University of Science and Technology (KAUST), Thuwal, Saudi Arabia; ^4^ Department of Medical Laboratory Sciences, College of Applied Medical Sciences, Majmaah University, Majmaah, Saudi Arabia

**Keywords:** human respiratory syncytial virus, hRSV, immunoinformatics, CTL epitope, HTL epitope, B-cell epitope, reverse vaccinology

## Abstract

**Background:**

Attempts to develop an hRSV vaccine have faced safety and efficacy challenges, with only three FDA-approved vaccines (Moderna’s Mresvia, Pfizer’s Abrysvo, and GSK’s Arexvy) available. These vaccines are limited to individuals over 60 years, require boosters, and only reduce disease severity without clearing the infection. Therefore, we employed a reverse vaccinology approach in this study to identify the most promising antigenic epitopes capable of eliciting a robust and protective immune response.

**Methodology:**

This study employed computational techniques to design a novel multi-epitope vaccine targeting hRSV. Using bioinformatics tools, candidate epitopes were identified from conserved viral proteins (F and G glycoproteins), assessing their immunogenicity, antigenicity, and allergenicity. Key tools included ExPASy, ProtParam, VaxiJen v2.0, AllergenFP v1.0, AllerTOP v2.0, NetCTL v1.2, IEDB, and Toxin-Pred. The vaccine construct was assessed for stability and toxicity through *in silico* analyses. We then characterized its kinetic properties, evaluated its structural integrity, and analyzed its interactions with Toll-like receptors (TLRs) using molecular docking, modeling, and refinement with AlphaFold3 and ClusPro.

**Results:**

The designed constructs showed strong antigenicity (0.5996 for F-based and 0.6048 for G-based vaccine), non-allergenicity, and stability (instability index <40). Among these, most amino acids were in the extracellular domain of the construct. Molecular docking and dynamics simulations indicated strong binding interactions with TLR1 and TLR4 and minimal RMSF fluctuations, which ensured structural stability. Strong humoral and cellular responses were suggested by in silico immune simulation demonstrating robust immune activation, with high levels of IgG, IgM, IL-2, and IFN-γ. The physical and chemical analyses revealed that the majority of amino acids from the F and G proteins were located in the extracellular domain of the construct. The presence of signal peptide cleavage sites in both glycoprotein components further facilitates antigen presentation to the immune system.

**Conclusions:**

This study presents a promising peptide-based vaccine candidate against hRSV that can effectively engage the immune system, showing strong immunogenicity and antigenicity. Future *in vitro* and *in vivo* studies are essential to evaluate the ability of the multi-epitope vaccine candidate to stimulate both humoral and cell-mediated immune responses and to assess its efficacy and safety profile.

## 1 Introduction

Human respiratory syncytial virus (hRSV) is a major cause of lower respiratory tract infections in infants, young children, and the elderly (1). It is estimated that hRSV causes 33 million new episodes of acute lower respiratory infections and 3.2 million hospital admissions annually worldwide (2). Despite the significant disease burden, currently, only three hRSV vaccines have been licensed by the FDA (3). Developing an effective hRSV vaccine has been challenging due to the complex immune responses to the virus and the risk of disease enhancement in vaccinated individuals (4).

Human RSV exhibits antigenic variability, with two major antigenic subgroups (A and B) circulating globally, complicating the design of a universal vaccine (4). Furthermore, previous attempts at hRSV vaccine development have been hampered by safety concerns, such as the phenomenon of vaccine-enhanced respiratory disease observed with a formalin-inactivated RSV vaccine candidate (5).

The most recent vaccine design efforts were focused on hRSV envelope proteins embedded in the lipid bilayer, specifically the attachment (G) glycoprotein and/or the fusion (F) glycoprotein. Human RSV G protein can exist in two forms, as complete membrane-bound glycoprotein (mG) that mediates viral attachment to host cells *in vivo* and secreted N-terminally truncated G protein (sG) (6). sG can modulate host immune responses, enabling it to evade, alter, or inactivate both innate defenses and the adaptive immune system, as well as influence the antiviral activity of monoclonal antibodies (mABs) (7). In addition, the extensive antigenic variability of G protein among different hRSV strains has been another significant obstacle to the development of an effective vaccine (8). Although these limitations were recently addressed to some extent through further optimization using the CsA adjuvant, these challenges shifted the focus to F glycoprotein, a more conserved viral surface component (9). The F protein allows hRSV penetration and fusion between adjacent cells to form syncytium. The viral F glycoprotein undergoes dynamic reconfiguration when binding to the target cell’s plasma membrane and thus exists in two forms: the prefusion form (pre-F) and the more stable post-fusion form or post-F (10). The unstable pre-F sequence was substantial in developing the two peptide-based vaccines and one mRNA vaccine approved by the FDA for hRSV, as it is highly immunogenic and stimulates the production of RSV-specific neutralizing antibodies (NAbs) (3).

BAFF and APRIL are crucial for B cell survival, differentiation, and antibody production. They interact with specific receptors on B cells, promoting their activation and proliferation, which is essential for generating a robust immune response. Previous studies have demonstrated that BAFF and APRIL can enhance the immunogenicity of vaccines. For instance, research has shown that plasmids expressing multimeric soluble BAFF or APRIL, when co-administered with other immunomodulatory agents, can significantly increase antibody titers and neutralizing antibody responses against HIV-1 (11, 12). Additionally, constructs combining HIV-1 envelope proteins with APRIL have been reported to enhance antibody responses in animal models (11). This has led to interest in their potential as adjuvants to improve vaccine efficacy while modulating immune responses.

The traditional approach of vaccine development relies on virus culturing and its activation which raises several safety concerns. Reverse vaccinology offers a promising approach and a rapid, cost-effective, and reliable methodology for the preliminary selection and design of novel multi-epitope vaccine candidates against hRSV. This approach involves comprehensive *in silico* analysis of the hRSV attachment and fusion proteome to identify the most promising antigenic epitopes capable of eliciting a robust and protective immune response (13, 14). Computational vaccinology techniques, such as epitope prediction, antigenicity and allergenicity analysis, and molecular docking, can be employed to design and evaluate multi-epitope vaccine candidates *in silico* before experimental validation (13, 14). By targeting multiple conserved epitopes from Glycoprotein and fusion hRSV proteins, a multi-epitope vaccine has the potential to provide broad coverage against both major hRSV subgroups and induce a balanced, long-lasting immune response.

The study aims to investigate the potential of a computationally designed multi-epitope vaccine to initiate a protective immune response, ensure the epitopes have stability and non-allergenicity, and provide broader coverage across the subtypes of hRSV. Furthermore. This study utilized a reverse vaccinology strategy to systematically analyze the hRSV proteome, identify immunogenic epitopes, and design a multi-epitope vaccine candidate against human RSV. The selected epitopes were further evaluated for their antigenicity, immunogenicity, allergenicity, and molecular docking properties to ensure the development of a safer vaccine formulation.

## 2 Materials and methods

### 2.1 Protein sequences retrieval

The amino acid sequences of fusion glycoprotein (F) [Human Orthopneumovirus] and attachment G protein [Human Respiratory Syncytial Virus A] with EMBL IDs QID88623.1 and ALB35397.1 respectively were retrieved from UniProt (15) in FASTA format to predict T cell, B cell, and IFN-gamma inducing epitopes.

### 2.2 Analysis of physicochemical properties

To calculate the chemical and physical properties of the target protein sequence, the ExPASy ProtParam tool (16) was used. This tool enables the calculation of a range of physicochemical parameters for proteins either retrieved from UniProtKB or provided as user-entered sequences In this study, the amino acid sequence of the target protein, represented in one-letter code, was input into the appropriate field, and the compute parameters option was selected. No additional data was required for the analysis. The computed metrics included molecular weight, aliphatic index, theoretical isoelectric point (pI): Determines the pH at which the protein has no net charge, instability index, extinction coefficient, grand average of hydropathicity grand average of hydropathy (GRAVY), and atomic composition.

### 2.3 Evaluation of antigenic properties

The potential vaccine candidates (PVCs) from the proteome of hRSV were predicted using the VaxiJen v2.0 server (17). VaxiJen is a Perl-based server with an HTML interface that classifies proteins as “Probable Non-Antigen” or “Probable Antigen” based on their antigen probability, which is expressed as a percentage. The default threshold value of 0.4 was used for this analysis. Additionally, two other tools were employed to distinguish between allergens and non-allergens: AllergenFP v.1.0 (18) and AllerTOP v.2.0 server (14). To predict the presence of transmembrane helices and signal peptides, TMHMM v2.0 (19) and SignalP 6 (20) tools were used, respectively. In all these tools the input parameters were protein sequences.

### 2.4 CTL epitope prediction and binding affinity analysis with MHC I allele

The prediction of cytotoxic T lymphocyte (CTL) epitopes was carried out using the NetCTL v1.2 server. The NetCTL 1.2 server predicts CTL epitopes in protein sequences (21) and an Immune Epitope Database (IEDB) tool (22) The input parameters consisted of peptide sequences, while the expected outputs included predicted CTL epitopes and binding affinities for MHC class I and II. These epitopes were classified, based on their binding to various major histocompatibility complex (MHC) alleles, including HLA-I, HLA-II/H-2-IAb, HLA-II/H-2-IAd, and H-2-Db. The CTL epitopes binding to HLA-I and H-2-Db alleles were retrieved from the NetCTL tool, while the CTL epitopes binding to H-2-IAb and HLA-II/H-2-IAd were obtained from the IEDB tool. Epitopes with a consensus score of less than 2 were considered excellent binders and selected for further analysis. The selected epitopes were then assessed for their antigenicity, immunogenicity, allergenic profile, and toxicity using the VaxiJen v.2.0, IEDB, AllergenFP v.1.0, and ToxinPred servers, respectively. The best epitopes were those with high antigenicity, non-allergenicity, and non-toxicity.

### 2.5 HTL epitope prediction and binding affinity analysis with MHC2 allele

The prediction of helper T lymphocyte (HTL) epitopes was performed using the NetMHCII pan 3.287 server (23). The HTL epitopes were classified based on their binding to human leukocyte antigen (HLA) class II alleles, specifically HLA-II/H-2-IAb and HLA-II/H-2-IAd. For the NetMHCII tool, the input parameters consisted of peptide sequences, while the expected outputs included predicted HTL epitopes and binding affinities. The antigenicity of each predicted epitope was evaluated using the VaxiJen v.2.0 server, with a threshold value of 0.4. To exclude potential allergenic epitopes, the AllergenFP v.1.089 server was employed. Furthermore, PyMOL was used to visualize the location of the predicted epitopes on the glycoprotein structure. Finally, the ToxinPred server was utilized to assess the toxicity profile of the selected epitopes. ToxinPred is a web server designed to predict whether proteins or peptides are toxic or non-toxic. We used peptide sequences as input parameters, and the expected output is a toxicity score indicating whether the peptide is toxic or non-toxic. The best epitopes were those that demonstrated high antigenicity, non-allergenicity, and non-toxicity after the filtration process.

### 2.6 Prediction of B-cell epitopes

The Immune Epitope Database (IEDB) (24) was employed to predict B cell epitopes based on the protein sequence. To select the final B cell epitope candidates, several servers were utilized to screen their properties: VaxiJen v.2.0, AllergenFP v.1.0, and ToxinPred. The best B cell epitope candidates were those that demonstrated high antigenicity, non-allergenicity, and non-toxicity after the screening process using these complementary computational tools.

### 2.7 Prediction of interferon-gamma-inducing epitopes

To predict and design IFN-γ epitopes for vaccine development, an IFN epitope web server was employed. This server enables users to predict and design peptides that induce IFN-gamma, MHC Class II bindings, or T-cell epitopes. We input peptide sequences as parameters, and the anticipated output includes both IFN-inducing and non-inducing epitopes. This server features three primary modules: Predict, Design, and Scan. It utilizes a dataset to classify IFN-γ epitopes into two distinct categories: those capable of producing IFN-γ and those that cannot. The server’s predictions are based on three strategies: hybrid, motif-based, and machine-learning approaches, thus offering an accuracy of up to 81.39% (25). For this study, multiple peptide sequences were input into the server, and the IDEB database, an experimentally validated dataset comprising 10,433 T-cell epitopes, was employed. Upon protein input, the hybrid approach combining motifs and support vector machines was selected to perform the predictions. The output was generated as numerical scores, where a positive value indicated the secretion of IFN-γ by the predicted epitopes.

### 2.8 Population coverage analysis

The population coverage analysis of human MHC alleles (HLA I and II) was carried out using the IEDB population coverage tool and the results were plotted in the form of a bar chart (26). In the study, default settings were used, and population coverage was evaluated for each class of MHC.

### 2.9 Multi-epitope vaccine design

To construct our vaccine, commonly used linker sequences in multi-epitope vaccine designs were employed to connect different types of epitopes. Linkers are an essential component in the design of multi-epitope vaccines, serving several crucial functions that enhance vaccine efficacy. They facilitate the proper folding of individual epitopes, ensuring that each maintains its correct conformation during protein synthesis, which is vital for effective recognition by the immune system. Additionally, linkers improve the overall immunogenicity of the vaccine by providing flexibility between epitopes, allowing for better presentation to immune cells and thereby enhancing the immune response. They also prevent steric hindrance that could occur if epitopes are positioned too closely together, ensuring effective interaction with T cell receptors and other components of the immune system. Furthermore, the incorporation of linkers contributes to the stability of the vaccine construct, helping to protect the epitopes from degradation. Thus, the strategic use of linkers is fundamental in optimizing the performance of multi-epitope vaccines Cytotoxic T lymphocyte (CTL) epitopes were linked using the AYY linker sequence. The AYY linker is a flexible linker that facilitates the proper folding and presentation of the CTL epitopes (27). Helper T lymphocyte (HTL) epitopes were connected using the GPGPG linker. This linker is commonly used to separate distinct epitopes while maintaining their structures and functions. The selected epitopes targeting B cells were linked using the KK linker. The KK linker, composed of two lysine residues, enhances the immunogenicity of the B cell epitopes by promoting their proper folding and exposure. BAFF and April adjuvant were then incorporated into the vaccine construct. The adjuvants were linked to the N-terminus of the vaccine sequence using the EAAAK linker. The EAAAK linker is a rigid alpha-helical linker that maintains the structural integrity and functionality of the adjuvant. The use of these specific linker sequences aims to optimize the presentation and immunogenicity of the different epitope types (CTL, HTL, and B cell) within the multi-epitope vaccine construct. The BAFF and April adjuvant, when linked to the vaccine, are expected to enhance the overall immune response generated by the vaccine.

### 2.10 Evaluation of physicochemical properties, antigenicity, and allergenicity of vaccine construct

The allergenic, antigenic, and toxicity profiles of the final multi-epitope vaccine construct were evaluated using the same computational tools identified above for the F and G protein epitopes. The VaxiJen v2.0 server was used to predict the antigenicity of the multi-epitope vaccine construct, the AllergenFP v1.0 server was employed to assess the allergenic potential of the multi-epitope vaccine, and the ToxinPred server was utilized to evaluate the toxicity profile of the multi-epitope vaccine construct.

### 2.11 Prediction of secondary structure

The three-dimensional structures of Toll-like receptor 2 (TLR2) and Toll-like receptor 4 (TLR4) were retrieved from the RCSB Protein Data Bank (PDB) database (28). These PDB structures served as the structural templates for the computational analysis of the vaccine construct. The secondary structure properties of the multi-epitope vaccine construct were determined using the Self-Optimized Prediction Method with Alignment (SOPMA) server and the Protein Structure Prediction Server (PSIPRED) v4.0 tool. For the SOPMA analysis, the default parameters were used.

### 2.12 Protein structural modeling, docking, refinement, and validation

The ERRAT server (29) was used to assess the overall quality of the 3D vaccine model by evaluating the statistics of non-bonded interactions between different atom types. To further refine the modeled structure, a CASP10 web-based approach, the Galaxy Refine tool (30) was utilized. The three-dimensional structures of the vaccine candidates were modeled using the Alphafold3 webserver following the standard settings (31). The TLR1 toll-like receptor sequence for Mus musculus was retrieved using the UniProt database (Uniprot ID-B9EJ46). The structure for the TLR1 receptor was also predicted using the Alphafold3 server. ClusPro molecular docking algorithm with no restraints or modifications in the structure (32) was used to perform molecular docking of the TLR domain with the vaccine candidates from both F and G glycoprotein (33). The dynamics and refinement studies were performed using Cabs-Flex 2.0 standalone (30) in the SS2 mode settings with a minimum distance along the protein chain was set at 3. The minimum length of restraints was set to 3.8 Å and the maximum length to 8.0Å. The number of cycles was increased to 100,000 at a temperature of 310K, while cycles between trajectories were set to 100, due to the large complex formed between TLR and vaccine candidate to have the best quality output per frame. A random seed was generated for every run for better comparison and correct error calculation. The interaction between the TLR receptor and vaccine candidate was analyzed by eye using Discovery Studio 2020 (34) and PyMOL (www.pymol.org) (35). The graphs for the fluctuation were plotted using Prism 10 (www.graphpad.com). To build the three-dimensional (3D) structures of the cytotoxic T-lymphocyte (CTL) and helper T-lymphocyte (HTL) epitopes within the vaccine construct, the PEPFOLD 3.5 web server was utilized (36).

### 2.13 In silico immune simulation

To model the immune response and assess the immunogenicity of the ALV vaccine in the host, we utilized the C-ImmSim server (https://kraken.iac.rm.cnr.it/C-IMMSIM/). The server can define a set of different models in one software that analyses both humoral and cellular responses including B-cells. The input parameters consisted of random seed, simulation volume, and simulation steps, while the expected outputs included parameters to configure the immune simulation, controlling randomness, size, and duration of the simulation. For this study, we configured the following parameters: Random Seed = 12,345, Simulation Volume = 10, and Simulation Steps = 1000. All other simulation parameters were maintained at their default settings to ensure consistency and reliability in the results.

## 3 Results

### 3.1 Protein sequences retrieval

The amino acid sequences of the RSV fusion (F) and attachment (G) glycoproteins were obtained from the UniProt knowledge base (UniProt Consortium, 2021). The F glycoprotein sequence consisted of 574 amino acid residues, while the attachment G glycoprotein sequence was 321 amino acids long. The molecular weights of the F and G glycoproteins were calculated to be 63,751 Daltons and 35,191 Daltons, respectively, based on their amino acid compositions. This information was also retrieved from the UniProt database (**Supplementary Table S1**).

### 3.2 Analysis of physicochemical properties of proteins

The physical and chemical characteristics of the RSV F and G glycoproteins were thoroughly analyzed to gain insights into their structural and functional properties. (**Supplementary Table S2**). The F glycoprotein, consisting of 574 amino acid residues, had a calculated molecular weight of 63,750.57 Daltons. The theoretical isoelectric point (pI) of the F protein was determined to be 9.13, indicating its basic nature. The grand average of hydropathicity (GRAVY) value, which represents the overall hydrophobicity/hydrophilicity of the protein, was -0.038, suggesting a slightly hydrophilic character. The aliphatic index, a measure of the relative volume occupied by aliphatic side chains, was found to be 102.18 for the F protein, indicating a relatively compact structure. The instability index was calculated to be 41.81, however, suggesting that the F protein may be unstable under certain conditions. The estimated coefficient value, a parameter used to predict the expression level of the protein, was determined to be 50,155. In contrast, the RSV G glycoprotein, with 321 amino acids, had a lower molecular weight of 35,190.86 Daltons. The pI value of the G protein was slightly higher than that of the F protein, at 9.77, reinforcing its basic character. Interestingly, the GRAVY value of the G protein was -0.636, indicating a more hydrophilic nature compared to the F protein. The aliphatic index and estimated coefficient values of the G protein were much lower than those of the F protein, at 68.38 and 20,190, respectively. However, the G protein was found to be more stable, with an instability index of 35.70, suggesting it may be less prone to degradation under various conditions.

### 3.3 Analysis of antigenicity and allergenicity of proteins

The RSV F and G glycoproteins were further analyzed to investigate their antigenic, allergenic, and toxic characteristics. Antigenic potential was assessed using a predictive algorithm, which measured the likelihood of a protein being recognized as an antigen. The F glycoprotein exhibited an antigenic score of 0.5295, while the G glycoprotein had a score of 0.5771. Both values exceeded the commonly used threshold of 0.4, indicating that these RSV glycoproteins possess significant antigenic properties. To evaluate the potential allergenicity of the F and G proteins, appropriate prediction models were employed. The analysis revealed that neither the F nor the G glycoprotein exhibited characteristics associated with allergenic proteins. This suggests that these RSV proteins are unlikely to elicit allergic responses. The proteins were also evaluated for potential toxic effects. The assessment did not identify any toxicity-related features within the amino acid sequences of the F and G glycoproteins. The findings from these bioinformatics analyses indicate that the RSV fusion (F) and attachment (G) glycoproteins have strong antigenic potential, which may contribute to their ability to stimulate immune responses. The absence of predicted allergenic and toxic properties suggests that these viral proteins are unlikely to cause adverse reactions or toxicity in the host.

### 3.4 CTL epitope prediction and binding affinity analysis with MHC I allele

The RSV F and G glycoproteins were further analyzed to identify specific epitopes with desired immunological and safety properties. The selected epitopes were evaluated for their antigenic, immunogenic, allergenic, and toxic characteristics. For the F protein, the following HLA-I class epitopes were chosen for detailed analysis: LTLAINALY, LSALRTGWY, and YTSVITIEL. All three epitopes exhibited antigenic and immunogenic properties and were found to be non-toxic (**Table 1**). However, only the LSALRTGWY epitope was predicted to be non-allergenic. Similarly, the G protein HLA-I class epitopes selected were: LLFISSCLY, SQVHTTSEY, and TTSQSTTIL. These G protein epitopes were identified as potential allergens. The SQVHTTSEY epitope was determined to be both immunogenic and antigenic. Further analysis focused on F protein epitopes for the H-2-IAd MHC class. The selected epitopes were: MELLIHRSSAIFLTL, LLIHRSSAIFLTLAI, and ELLIHRSSAIFLTLA. All three epitopes demonstrated immunogenic, antigenic, and non-toxic properties. Only the ELLIHRSSAIFLTLA epitope was predicted to be an allergen, while the other two were classified as non-allergenic.

**Table 1:** List of overall attributes of MHC class I interacting CTL epitopes that were employed for designing a vaccine construct.

MHC-I Allele	Epitope	Protein	Length	Immunogenicity score	Antigenicity Score	Allergenicity	Toxicity
HLA1	LTLAINALY	Fusion Glycoprotein	9	0.18582	0.7147	Allergen	Non-Toxin
	LSALRTGWY	Fusion Glycoprotein	9	0.2465	1.1132	Non-allergen	Non-Toxin
	YTSVITIEL	Fusion Glycoprotein	9	0.3248	0.6842	Allergen	Non-Toxin
	LLFISSCLY	Attachment Glycoprotein	9	-0.19689	0.1194	Allergen	Toxin
	SQVHTTSEY	Attachment Glycoprotein	9	0.03917	0.6339	Allergen	Non-Toxin
	TTSQSTTIL	Attachment Glycoprotein	9	-0.1843	0.3578	Allergen	Non-Toxin
HLA II/H-2-IAd	MELLIHRSSAIFLTL	Fusion Glycoprotein	15	0.16576	0.4136	Non-allergen	Non-Toxin
	LLIHRSSAIFLTLAI	Fusion Glycoprotein	15	0.12489	0.6449	Non-allergen	Non-Toxin
	ELLIHRSSAIFLTLA	Fusion Glycoprotein	15	0.18756	0.4213	Allergen	Non-Toxin
	NLKSIAQITLSILAM	Attachment Glycoprotein	15	-0.04729	0.9619	Allergen	Non-Toxin
	KLNLKSIAQITLSI	Attachment Glycoprotein	14	-0.26934	1.1468	Non-allergen	Non-Toxin
	LYKLNLKSIAQITLS	Attachment Glycoprotein	15	-0.2629	0.9181	Allergen	Non-Toxin
HLA II/H-2-IAb	FYQSTCSAVSRGYLS	Fusion Glycoprotein	15	-0.38997	0.5709	Non-allergen	Non-Toxin
	GVGSAIASGIAVSKV	Fusion Glycoprotein	15	-0.13683	0.6023	Non-allergen	Non-Toxin
	TREFSVNAGVTTPLS	Fusion Glycoprotein	15	0.1929	0.3074	Non-allergen	Non-Toxin
	IAAIIFIASANHKVT	Attachment Glycoprotein	15	0.30119	0.6127	Non-allergen	Non-Toxin
	AIIFIASANHKVTLT	Attachment Glycoprotein	15	0.08604	0.7845	Allergen	Non-Toxin
	IIFIASANHKVTLTT	Attachment Glycoprotein	15	-0.00772	0.6941	Non-allergen	Non-Toxin
H-2-Db	YMLTNSELL	Fusion Glycoprotein	9	-0.04855	0.2930	Non-allergen	Non-Toxin
	VSLSNGVSV	Fusion Glycoprotein	9	-0.20629	0.8926	Allergen	Non-Toxin
	LAMIISTSL	Attachment Glycoprotein	9	-0.01311	0.5518	Allergen	Non-Toxin
	AMIISTSLI	Attachment Glycoprotein	9	-0.09354	0.3295	Allergen	Non-Toxin

The G protein H-2-Iad MHC class epitopes examined were: NLKSIAQITLSILAM, KLNLKSIAQITLSIL, and LYKLNLKSIAQITLS. These epitopes were found to be antigenic and non-toxic, but non-immunogenic. NLKSIAQITLSILAM and LYKLNLKSIAQITLS were identified as potential allergens. For the H-2-Iab MHC class, the selected F protein epitopes were: FYQSTCSAVSRGYLS, GVGSAIASGIAVSKV, and TREFSVNAGVTTPLS. All these epitopes were determined to be non-toxic and non-allergenic. However, only the TREFSVNAGVTTPLS epitope was antigenic, while FYQSTCSAVSRGYLS and GVGSAIASGIAVSKV were non-immunogenic. The G protein H-2-Iab MHC class epitopes analyzed were: IAAIIFIASANHKVT, AIIFIASANHKVTLT, and IIFIASANHKVTLTT. These epitopes exhibited antigenic and non-toxic properties, and IAAIIFIASANHKVT and AIIFIASANHKVTLT were also immunogenic. Lastly, for the H-2-Db MHC class, the F protein epitopes selected were: YMLTNSELL and VSLSNGVSV. Both epitopes were found to be non-toxic and non-immunogenic. However, the YMLTNSELL epitope was classified as non-antigenic and non-allergenic, while the VSLSNGVSV epitope was antigenic but allergenic. The G protein H-2-Db MHC class epitopes examined were LAMIISTSL and AMIISTSLI. These epitopes were determined to be non-toxic and non-immunogenic but were predicted to be allergenic.

### 3.5 HTL epitope prediction and binding affinity analysis with MHC2 allele

RSV F and G glycoproteins were further analyzed to identify additional epitopes with desirable immunological and safety characteristics, focusing on the H-2-IAb and H-2-Iad MHC class contexts (**Table 2**). For the H-2-Iab MHC class, the following F protein epitopes were selected for analysis: GVGSAIASGIAVSKV, TREFSVNAGVTTPLS, and EFSVNAGVTTPLSTY. All three epitopes were found to be non-toxic and non-allergenic. However, only TREFSVNAGVTTPLS and EFSVNAGVTTPLSTY were determined to be immunogenic. The G protein epitopes examined for the H-2-Iab MHC class were: IAAIIFIASANHKVT, IIFIASANHKVTLTT, and AIIFIASANHKVTLT. All three of these epitopes exhibited antigenic, non-toxic, and non-allergenic properties. For the H-2-Iad MHC class, the selected F protein epitopes were: MELLIHRSSAIFLTL, GVGSAIASGIAVSKV, and AIASGIAVSKVLHLE. All of these epitopes were found to be antigenic, non-toxic, and non-allergenic. The G protein epitopes analyzed for the H-2-Iad MHC class were NLKSIAQITLSILAM, AAIIFIASANHKVTL, and KLNLKSIAQITLSIL. These epitopes were all identified as antigenic and non-toxic. However, only the NLKSIAQITLSILAM epitope was predicted to be an allergen, while AAIIFIASANHKVTL and KLNLKSIAQITLSIL were classified as non-allergenic.

**Table 2:** List of overall attributes of MHC class II interacting HTL epitopes used for designing a vaccine construct.

MHC-II Allele	Epitope	Protein	Length	Immunogenicity Score	Antigenicity Score	Allergenicity	Toxicity
HLA II/H-2-IAb	GVGSAIASGIAVSKV	Fusion Glycoprotein	15	-0.13683	0.6023	Non-allergen	Non-Toxin
	TREFSVNAGVTTPLS	Fusion Glycoprotein	15	0.1929	0.3074	Non-allergen	Non-Toxin
	EFSVNAGVTTPLSTY	Fusion Glycoprotein	15	0.03026	0.2190	Non-allergen	Non-Toxin
	IAAIIFIASANHKVT	Attachment Glycoprotein	15	0.30119	0.6127	Non-allergen	Non-Toxin
	IIFIASANHKVTLTT	Attachment Glycoprotein	15	-0.00772	0.6941	Non-allergen	Non-Toxin
	AIIFIASANHKVTLT	Attachment Glycoprotein	15	0.08604	0.7845	Allergen	Non-Toxin
HLA II/H-2-IAd	MELLIHRSSAIFLTL	Fusion Glycoprotein	15	0.16576	0.4136	Non-allergen	Non-Toxin
	GVGSAIASGIAVSKV	Fusion Glycoprotein	15	-0.13683	0.6023	Non-allergen	Non-Toxin
	AIASGIAVSKVLHLE	Fusion Glycoprotein	15	-0.23339	0.8407	Non-allergen	Non-Toxin
	NLKSIAQITLSILAM	Attachment Glycoprotein	15	-0.04729	0.9619	Allergen	Non-Toxin
	AAIIFIASANHKVTL	Attachment Glycoprotein	15	0.21554	0.6395	Non-allergen	Non-Toxin
	KLNLKSIAQITLSIL	Attachment Glycoprotein	15	-0.23436	1.1468	Non-allergen	Non-Toxin

### 3.6 Prediction Of B-cell epitopes

The IEDB (Immune Epitope Database) server was utilized to analyze the F and G protein and identify potential epitopes. The epitopes that exceeded the 0.5 threshold were then evaluated for their allergenicity, antigenicity, immunogenicity, and toxicity characteristics. The epitope that exhibited the highest score in the IEDB analysis was deemed the most promising candidate for further study. Based on the comprehensive evaluation using the IEDB server, a subset of F and G protein epitopes was selected for further analysis due to their potential to induce a B-cell response (SF1 and SF2). The selected F protein epitopes were ETKCNGTDT, KCTASNKN, and NTPVTLS. All three were found to be antigenic. However, only the NTPVTLS epitope was determined to be both immunogenic and non-toxic, making it the most promising candidate from this group for further investigation. Similarly, a set of G protein epitopes was selected for analysis: LSGTTSQST, MSKTKDQRTAKT, and TNQIKNTTPTYLTQN. All three G protein epitopes were identified as antigenic and non-toxic. However, none were predicted to be immunogenic (**Table 3**).

**Table 3:** List of overall attributes of B cell epitopes that were employed to design a vaccine construct.

Protein	Epitope	Length	Immunogenicity	Antigenicity	Allergenicity	Toxicity
Fusion Glycoprotein	ETKCNGTDT	9	-0.05293	0.9375	Non-allergen	Toxin
	KCTASNKN	8	-0.29722	1.2880	Allergen	Toxin
	NTPVTLS	7	0.0653	1.1172	Allergen	Non-Toxin
Attachment Glycoprotein	LSGTTSQST	9	-0.26229	0.7385	Non-allergen	Non-Toxin
	MSKTKDQRTAKT	12	-0.33818	0.6219	Non-allergen	Non-Toxin
	TNQIKNTTPTYLTQN	15	-0.06634	0.5845	Allergen	Non-Toxin

### 3.7 Prediction of interferon-gamma-inducing epitopes

In addition to the B-cell response-inducing epitopes, the analysis also identified a set of interferon-gamma-inducing F protein epitopes that were selected for further investigation. These epitopes were LPIGAVSIVAIALLL, IGAVSIVAIALLLRL, and PIGAVSIVAIALLLR. All three were found to be antigenic, immunogenic, and non-toxic, making them promising candidates for inclusion in a multi-epitope vaccine construct. The analysis also identified a single G protein epitope, TNQIKNTTPTYLTQN, that was also selected for further consideration (**Table 4**).

**Table 4:** List of overall attributes of Interferon-Gamma inducing F and G glycoprotein epitopes that were employed to design a vaccine construct.

Protein	Epitope	Position	Immunogenicity	Antigenicity	Allergenicity	Toxicity
Fusion Glycoprotein	LPIGAVSIVAIALLL	15	0.35299	1.1321	Non-allergen	Non-Toxin
	IGAVSIVAIALLLRL	15	0.26838	1.1321	Non-allergen	Non-Toxin
	PIGAVSIVAIALLLR	15	0.27879	1.0998	Allergen	Non-Toxin
Attachment Glycoprotein	TNQIKNTTPTYLTQN	15	-0.06634	0.5845	Allergen	Non-Toxin

### 3.8 Multi-epitope vaccine design

**Figure 1:**
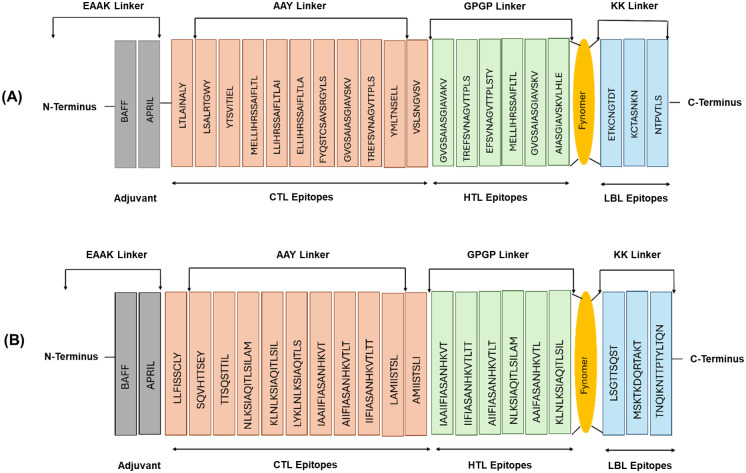
Multi-epitope vaccine construct of F glycoprotein **(A)** and G glycoprotein **(B)** with epitopes linked by different linkers. The orange color shows CTL epitopes interconnected by AAY linkers, the green color represents HTL epitopes interconnected by GPGP linkers, and the blue color shows LBL epitopes interconnected by KK linkers. BAFF and APRIL adjuvants are connected to the N-terminus via EAAK linkers.

Based on the detailed analysis and evaluation of the F and G protein epitopes, a multi-epitope vaccine construct was designed that met the criteria for antigenicity, allergenicity, toxicity, and population coverage. The final vaccine construct comprised a sequence of 315 amino acid residues, which incorporated non-overlapping epitopes selected from the F protein. The vaccine design included 11 cytotoxic T lymphocyte (CTL) epitopes, six helper T lymphocyte (HTL) epitopes, and three B-cell-inducing epitopes. To facilitate the appropriate presentation and processing of the different epitope types, specific linker sequences were utilized to connect the epitopes within the multi-epitope vaccine construct. The CTL epitopes were joined using Ala-Ala-Tyr (AAY) linkers, which are known to enhance CD8+ T cell activation and antigen processing. The HTL epitopes were connected by Gly-Pro-Gly-Pro-Gly (GPGPG) linkers, a flexible linker sequence that allows for optimal presentation of the helper T cell epitopes. The B-cell-inducing epitopes were linked using KK Lys-Lys linkers, which have been shown to improve B-cell recognition and antibody production. The strategic arrangement of the F protein’s different epitope types, along with the incorporation of the selected linker sequences, is depicted in **Figure 1A**. Similarly, a 317 amino acid residue long G protein multi-epitope vaccine construct included 11 cytotoxic T lymphocyte (CTL) epitopes, 6 helper T lymphocyte (HTL) epitopes, and 3 B-cell-inducing epitopes. The same linker strategies used for the F protein epitopes were also applied to the G protein epitopes to facilitate appropriate presentation and processing. Specifically, the CTL epitopes were joined using AAY linkers, the HTL epitopes were connected by GPGPG linkers, and the B-cell-inducing epitopes were linked using KK linkers. The visual representation of the multi-epitope vaccine construct, including the arrangement and linkage of the epitopes from G protein, is shown in **Figure 1B**.

### 3.9 Evaluation of physical properties of vaccine construct

After constructing the multi-epitope vaccines based on the F and G proteins, their physicochemical properties were determined and compared with the original F and G protein values reported in (**Supplementary Table S3**). The analysis revealed that the number of amino acids in the vaccine constructs was reduced to 315 and 317 for the F and G protein-based vaccines respectively, compared to the original full-length protein sequences. The molecular weight of the F protein-based vaccine construct decreased from 63,750.57 Da to 31,982.84 Da, while the G protein-based vaccine showed a slight decrease from 35,190.86 Da to 33,292.41 Da. The theoretical isoelectric point (pI) values of the F and G protein-based vaccine constructs were calculated to be 9.46 and 10.07, respectively. The grand average of hydropathicity (GRAVY) values for the F and G protein-based vaccine constructs were positive, at 0.448 and 0.598 respectively, indicating that the proteins are generally hydrophobic. The aliphatic index, which provides an estimate of the relative volume occupied by aliphatic side chains, was higher for the vaccine constructs compared to the original proteins, with values of 101.40 for the F protein-based vaccine and 117.22 for the G protein-based vaccine. The instability index, which predicts the stability of a protein, was less than 40 for both the F and G protein-based vaccine constructs, at 24.42 and 20.22, respectively, suggesting that both vaccine constructs are stable. The extinction coefficient values, which indicate the amount of light absorbed by a protein solution, were calculated to be 30,955 M−1 cm−1 for the F protein-based vaccine and 20,860 M−1 cm−1 for the G protein-based vaccine. The antigenic values for the F and G protein-based vaccine constructs were 0.5996 and 0.6048 respectively, which are higher than the threshold of 0.4, confirming their antigenic potential. Both the F and G protein-based vaccine constructs were also assessed to be non-allergenic. Overall, the physicochemical characterization of the multi-epitope vaccine constructs demonstrated favorable properties, including reduced molecular weight, improved stability, and retained antigenicity, compared to the original F and G proteins, indicating their suitability for further development and evaluation as potential respiratory syncytial virus vaccine candidates.

### 3.10 Evaluation of antigenic and allergenicity properties of the vaccine constructs

The antigenicity of the vaccine constructs derived from the F (fusion) and G (attachment) proteins was predicted using the default settings in the antigenicity prediction tool, with a threshold value of 0.4. The overall antigenicity prediction score for the F protein-based vaccine construct was 0.5996, while the score for the G protein-based vaccine construct was 0.6048. Both of these scores exceeded the 0.4 thresholds, indicating that the vaccine constructs derived from the F and G proteins were likely to be “Probable ANTIGENS”. This antigenicity analysis revealed that both the F and G protein-based vaccine constructs exhibited strong antigenic potential. Allergen and AllerTOP tools revealed that the vaccine against F protein and G protein was “Probable Non-Allergen” (Data not shown).

**Figure 2:**
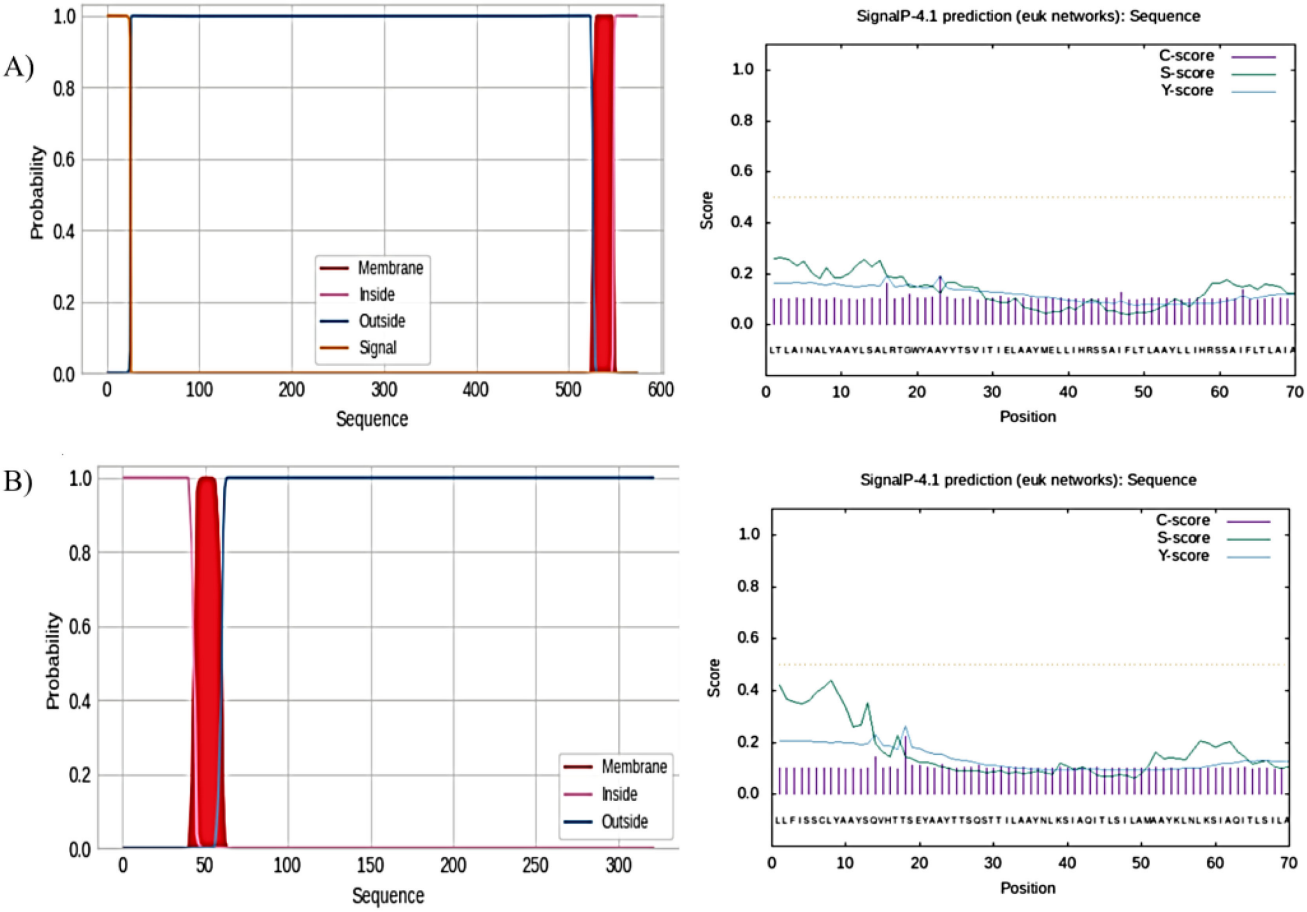
TMHMM server for the prediction of the nature of amino acid residues. **(A)** Nature of amino acid residues of F protein and prediction of the presence of signal peptide on F proteins. **(B)** Nature of amino acid residues of G protein and prediction of the presence of signal peptide on G proteins.

The TMHMM server was used to analyze the presence and distribution of transmembrane helices in the vaccine constructs, derived from the F and G glycoproteins. For the F glycoprotein vaccine construct, the analysis revealed the presence of two transmembrane helices. The amino acids were distributed as follows: 1) Outside region: Amino acids 1-19 and 78-315; transmembrane helices (purple); Amino acids 20-42 and 55-77. 2) Inside region (between transmembrane helices): Amino acids 43-54. This distribution indicated that the majority of the amino acids in the F protein-based vaccine construct were located in the outside region, which is the extracellular domain of the protein. The SignalP server was used to analyze the signal peptide and cleavage site predictions. For the F glycoprotein vaccine construct, the analysis revealed that the C-score showed a distinct peak at the 23rd amino acid position, indicating the predicted cleavage site. S-score (signal peptide score graph showed the presence of a signal peptide sequence. The Y-score which combines the C-score and S-score, also reached a maximum at the 23rd amino acid position, further confirming the predicted cleavage site. These results suggest that the F glycoprotein vaccine construct is likely to be cleaved at the 23rd amino acid position, resulting in the removal of the signal peptide and the presentation of the mature, processed form of the antigen (**Figure 2A**).

Similarly, the analysis of the G glycoprotein vaccine construct revealed the presence of five transmembrane helices. The amino acid distribution was as follows: 3) Outside region: Amino acids 1-31, 99-107, and 166-234; transmembrane helices (purple); amino acids 32-54, 76-98, 108-130, 143-165, and 235-257. 4) Inside region (between transmembrane helices): Amino acids 55-75, 131-142, and 258-317 Again, most of the amino acids in the G protein-based vaccine construct were in the outside region, which is the extracellular domain of the protein. For the G glycoprotein vaccine construct, the SignalP analysis showed that the Cleavage Site Score (C-score) peaked at the 18th amino acid position, indicating the predicted cleavage site. The Signal Peptide Score (S-score) graph suggested the presence of a signal peptide sequence. The Y-score also reached a maximum at the 18th amino acid position, corroborating the predicted cleavage site. These results suggest that the G glycoprotein vaccine construct is predicted to be cleaved at the 18th amino acid position, leading to the removal of the signal peptide and the exposure of the mature antigen (**Figure 2B**). We identified the signal peptide cleavage sites for both the F and G glycoprotein vaccine constructs. These signal peptide cleavage sites ensure the proper processing and presentation of the antigens to the immune system.

### 3.11 Prediction of secondary structure

The secondary structure of the F and G glycoprotein vaccine constructs was predicted, using the PSIPRED algorithm. For the F glycoprotein vaccine construct, the analysis revealed that the secondary structure composition contained 23.7% helices, 13.70% strands, and 34.67% coils. The results showed that the F protein-based vaccine construct is predominantly composed of coil regions, with a significant proportion of helical structures and a smaller fraction of beta-strand regions. The analysis of the G glycoprotein vaccine construct showed a similar trend, with the secondary structure dominated by helical elements, followed by coils and strands. A helical structure was observed in both the F and G protein-based vaccine constructs which play a crucial role in maintaining the native-like conformation of proteins and preserving the integrity of important functional epitopes (SF3 and SF4).

### 3.12 Three-dimensional structural modeling, interaction, and stability

We predicted the three-dimensional structure of the vaccine constructs namely, F1, F2, F3 for F-glycoprotein and G1, G2, G3 for G-glycoprotein. The structure modeling was performed using the latest artificial intelligence (AI) and machine learning (ML) based algorithm Alphafold3 webserver (31). The TLR1 toll-like receptor sequence for Mus musculus was retrieved using the UniProt database (Uniprot ID-B9EJ46). The structure for the TLR1 receptor was also predicted using the Alphafold3 server. All models obtained were of high quality with plDDT scores for all reported to be >70. Further, we wanted to test if the vaccine candidates would bind and, in turn, block the TLR receptors. Hence, the 3D models for all proteins (TLR and vaccine candidates) were then used to predict TLR: vaccine complexes. The protein: protein docking simulation models were performed using the ClusPro docking algorithm (32).

**Figure 3:**
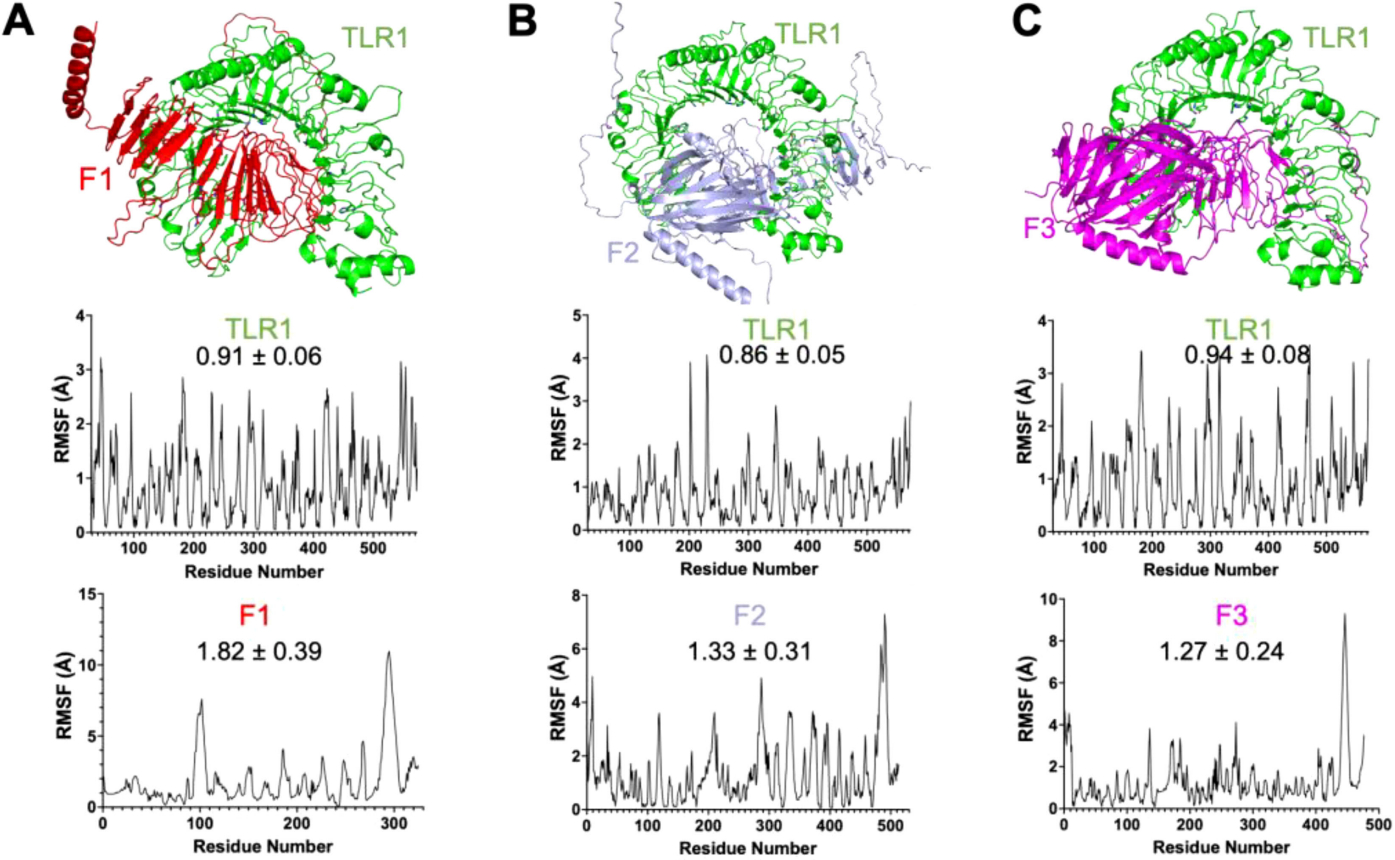
Three-dimensional protein complex between [**(A)**: Top] TLR1 domain (green) and F1 vaccine candidate (red), [**(B)**: Top] TLR1 domain (green) and F2 vaccine candidate (slate-blue) and [**(C)**: Top] TLR1 domain (green) and F3 vaccine candidate (magenta) shown in cartoon representation. Root mean square fluctuation (RMSF) plots for the interaction between TLR1 and vaccine candidates are below the respective structural representation arranged accordingly. The RMSF value is also mentioned on the plot with n=3. Interaction residues also mentioned in the Supplementary Table are shown as sticks.

**Figure 4:**
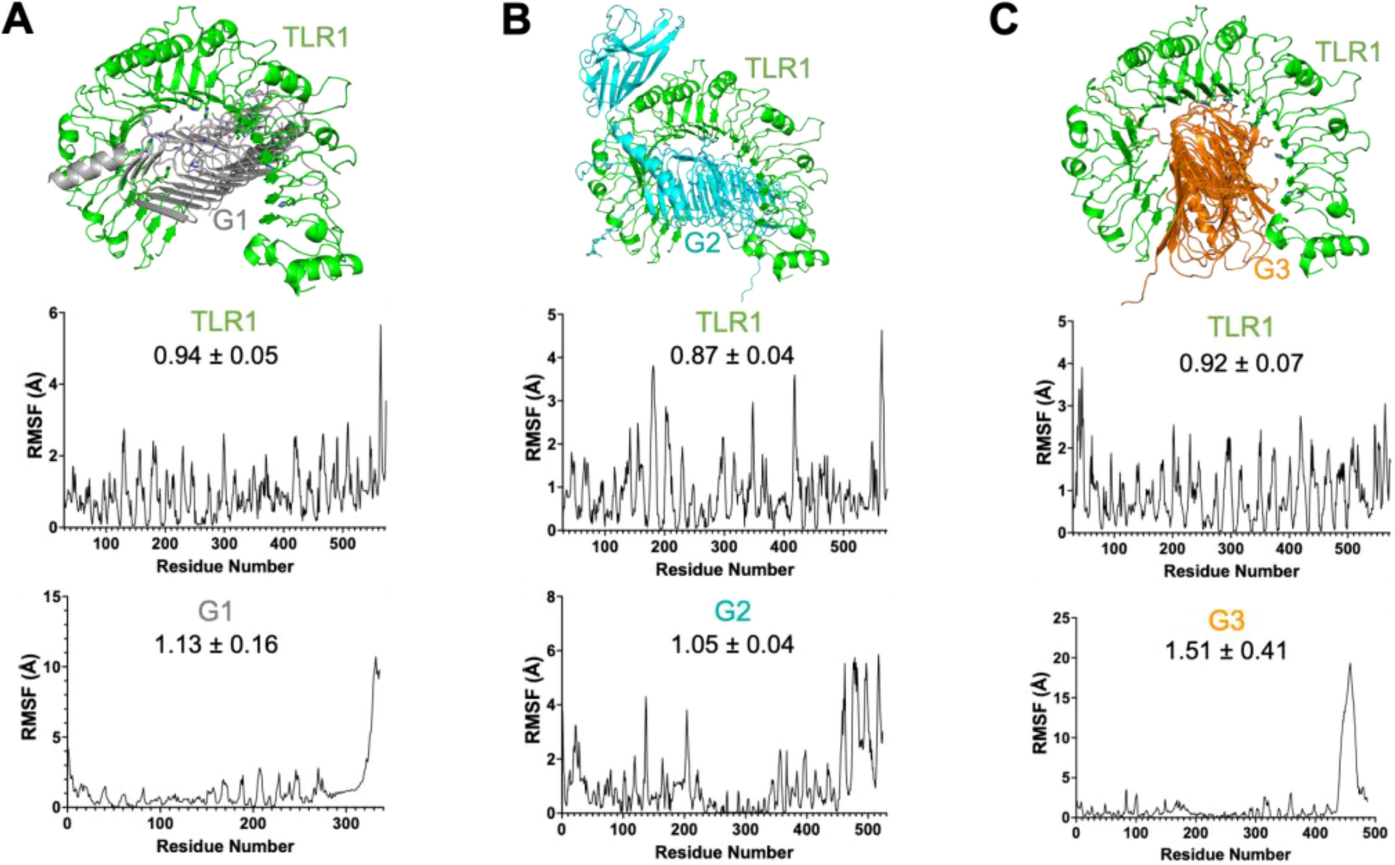
Three-dimensional protein complex between [**(A)**: Top] TLR1 domain (green) and G1 vaccine candidate (gray), [**(B)**: Top] TLR1 domain (green) and G2 vaccine candidate (cyan) and [**(C)**: Top] TLR1 domain (green) and G3 vaccine candidate (orange) shown in cartoon representation. Root means square fluctuation (RMSF) plots for the interaction between TLR1 and vaccine candidates are below the respective structural representation arranged accordingly. The RMSF value is also mentioned on the plot with n=3. Interaction residues also mentioned in the Supplementary Table are shown as sticks.

We found that all vaccine candidates could occupy the interaction binding pocket on the TLR receptor. The interactions were mostly charged where critical positive and negative amino acids formed the salt bridges and combined with pi-pi interaction through bulky hydrophobic residues. The interaction is shown for TLR1:F1 (**Figure 3A** top), TLR1:F2 (**Figure 3B** top), TLR1:F3 (**Figure 3C** top), TLR1:G1 (**Figure 4A** top), TLR1:G2 (**Figure 4B** top), TLR1:G3 (**Figure 4C** top). Important interaction residues found are listed in **Supplementary Table S4**.

We then verified the stability of the TLR:vaccine complex using dynamics and stability analysis using CabsFlex 2.0 standalone (30). We found the TLR: vaccine candidates to be very stable and had the per residue fluctuation (RMSF) within the required limits through time. The RMSF plots are shown for TLR1:F1 (**Figures 3B, C**, TLR1:F2 (**Figures 3B, C**), TLR1:F3(**Figures 3B, C**), TLR1:G1 (**Figures 4B, C**), TLR1:G2 (**Figures 4B, C**), TLR1:G3 (**Figures 4B, C**). Though all vaccine candidates were within the allowed RMSF limit, the best ones found were G1 and G2, with the least residue fluctuations. The higher fluctuations observed at the C-terminal ends of all the vaccine candidates are due to the extended unstructured region on the candidates, which includes the 6xHistag. Overall, we found all predicted vaccine candidates to be well-folded and specifically targeting the TLR domains.

### 3.13 Immune stimulation

**Figure 5:**
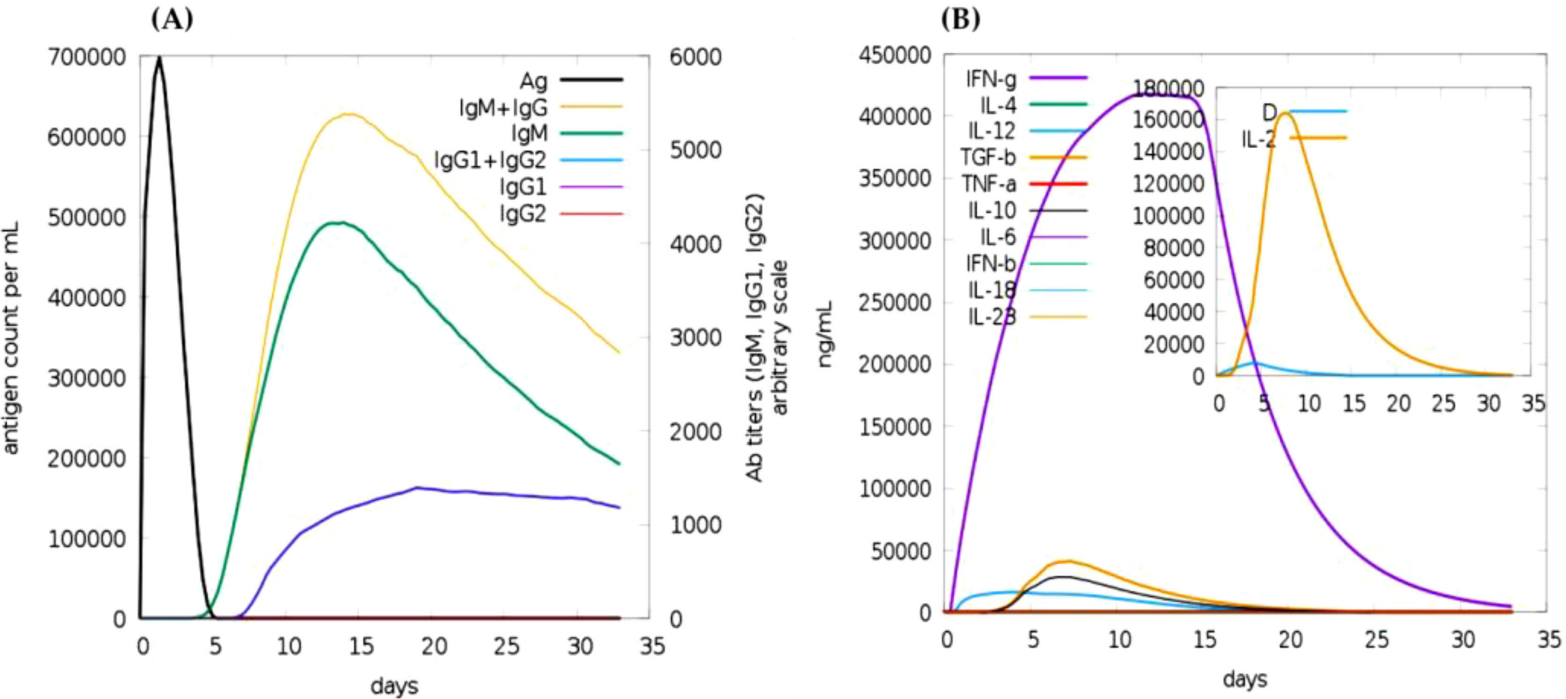
Immune simulation of the predicted vaccine following two injections via the c-immsim server. **(A)** Immunoglobulin production in response to antigen injections, with specific subclasses represented in different colors. **(B)** Cytokine secretion induced by the vaccine highlights IL-2 levels and a measure of diversity.

The immune simulation results showed a significant increase in the primary, secondary, and tertiary immune responses, corresponding with a reduction in antigen concentration (**Figure 5**). The levels of IL-2 were found to align with the measure of diversity, indicating a robust immune activation. Furthermore, an increase in diversity over time is interpreted as a danger signal, particularly in conjunction with the presence of leukocyte growth factor. Thus, a lower measure of diversity value reflects diminished immune diversity, suggesting potential implications for the effectiveness of the immune response.

## 4 Discussion

Over the past few years, significant resources and efforts have been dedicated to developing a safe and effective vaccine against hRSV, a major respiratory pathogen. Natural RSV infection fails to provide lasting immunity, leading to multiple infections throughout an individual’s life. Consequently, designing a vaccine that effectively mimics the immune response generated by natural RSV infection, while accounting for the variability among different viral strains remains a substantial challenge for researchers (37).

The emergence of diverse vaccine candidates utilizing various technologies presents an opportunity to tailor immunization strategies to meet the specific needs of vulnerable age groups. Arexvy^®^ (GSK) and Abrysvo^®^ (Pfizer) are significant advancements in this area, being the first vaccines approved to prevent hRSV infections in older adults (3). Notably, Abrysvo^®^ extends its utility by offering passive immunization for infants through maternal administration during pregnancy, thereby providing dual protection for both mothers and their newborns (38). Utilizing the mRNA platform, Moderna received U.S. FDA approval for the RSV vaccine mRESVIA(mRNA-1345). These approaches underscore the importance of developing age-specific vaccine strategies that can effectively address the unique immunological challenges faced by different populations (3, 38). These innovative vaccines pave the way for more personalized and effective immunization programs against hRSV (38).

Recent advancements in computational biology, immunoinformatics, and reverse vaccinology hold promise for accelerating the development of safe and effective vaccines in a more time- and cost-efficient manner (39, 40). By leveraging genomic and proteomic data, we can identify potential epitopes and design vaccines with immunogenic subunits that elicit long-lasting immunity, facilitating the validation of these candidates in preclinical settings (41, 42). In this study, we employ immunoinformatic approaches to identify key B cells, cytotoxic T lymphocyte (CTL), and helper T lymphocyte (HTL) epitopes derived from F and G proteins of hRSV, to develop a highly safe, synthetic multi-component vaccine tailored for the human host.

In this study, we employed several complementary tools to ensure a robust analysis of potential epitopes derived from the F and G glycoproteins of the hRSV. For Cytotoxic T Lymphocyte (CTL) epitope prediction, we utilized both the NetCTL v1.2 and the Immune Epitope Database (IEDB) tools. NetCTL focuses on predicting CTL epitopes based on their binding affinities to MHC class I molecules, providing a quantitative measure of potential immunogenicity. Meanwhile, IEDB offers additional validation through a comprehensive database of experimentally confirmed epitopes, enhancing the reliability of our findings. For Helper T Lymphocyte (HTL) epitope prediction, we employed NetMHCII pan 3.287 alongside IEDB, allowing us to cross-verify predictions and bolster confidence in the selected epitopes for further analysis. This dual approach is critical, as it ensures that our predicted HTL epitopes are not only computationally validated but also supported by empirical data. Additionally, for B-cell epitope prediction, we relied on IEDB for preliminary assessments while further investigating the epitopes through VaxiJen v2.0 and AllergenFP v1.0. This multifaceted approach enabled us to evaluate the antigenicity and allergenic potential of the identified epitopes comprehensively, laying a solid foundation for the design of a safe and effective multi-epitope vaccine against hRSV.

An emerging and important field of research is multi-epitope vaccines. Multi-epitope vaccinations offer the benefit of reducing undesirable effects, such as allergies and antigenic load. This results in a more specific immune response toward conserved epitopes without the reversion of the pathogenesis of the virus. The combined effect of the present epitopes from various antigens exceeds an isolated antigen epitope’s ability to stimulate an immune response, including both humoral and cell-mediated responses. Multi-epitope vaccinations have been developed to limit a diverse range of diseases (43). From a pharmacological perspective, multi-epitope vaccinations exhibit advantageous characteristics. Multi-epitope vaccines can be effectively and economically generated due to their focus on chemically well-characterized peptides. The multi-epitope vaccination can protect a broad spectrum of pathogens or different strains of a certain pathogen, particularly for highly adaptable pathogens that undergo many mutations and give rise to new variations (44).

The physicochemical characterization of F and G protein-based multi-epitope vaccines revealed a reduction in the size of 574-amino-acid-long F glycoprotein (molecular weight: 63,750.57 Da) to 315 amino acids (molecular weight: 31,982.84 Da), and 321-amino acid-long G glycoprotein (molecular weight: 35,190.86 Da) to 317 amino acids (molecular weight: 33,292.41 Da). The pI values of 9.46 and positive GRAVY value of 0.448 for F protein-based vaccine constructs, along with the pI value of 10.07, and positive GRAVY values of 0.598 for G protein-based vaccine constructs, indicated the basic and hydrophobic nature of these vaccines.

Additionally, the higher aliphatic index, with values above 100, and the instability index values below 40 for both the F and G protein-based vaccine constructs, highlight the significant relative volume occupied by aliphatic side chains and the stability of these protein-based vaccine constructs. These favorable physicochemical properties, compared to the original F and G proteins indicate their potential suitability for further development and evaluation as hRSV vaccine candidates. The findings of this research align with a previous study, which reported similar physiochemical stability in multi-epitope-based vaccine design against hRSV (45).

The F and G protein-based vaccine constructs demonstrated higher antigenicity, with values of 0.5996 and 0.6048, respectively. AllerTOP analysis further confirmed the non-allergic nature of the selected proteins for the hRSV vaccine. This antigenic and non-allergenic profile suggests that the vaccine constructs have the potential to stimulate an active immune response against the hRSV without triggering allergic reactions in humans. Consequently, these proteins are promising candidates for developing a vaccine against hRSV.

A similar study reported the antigenic and non-allergic properties of the multi-epitope vaccine candidates using the AntigenPro and Vaxijen servers (46). Furthermore, research on RSV on structural proteins, such as MHC II, 3 B-cell epitopes, and 6MHC-I revealed their antigenic and non-allergic nature. These characteristics demonstrated their ability to stimulate immune responses and prevent viral replication (47, 48). The distribution of transmembrane helices in the F and G glycoproteins was analyzed using the TMHMM server, revealing that the proteins consist of 315 and 317 amino acids, respectively. The F protein contains two transmembrane helices, with 256 of its amino acids in the extracellular region. In contrast, the G protein has five transmembrane helices, with 107 residues in its extracellular region. These results suggest that most amino acids in these protein-based vaccine constructs are located in the extracellular domains of the proteins. The presence of transmembrane helices was similarly reported in a previous study involving the TMHMM server, which identified two transmembrane helices in the envelope protein (49).

The secondary structure analysis of the F glycoprotein-based vaccine construct revealed a predominant composition of coil regions (34.67%), followed by a significant proportion of helical structures (23.7%) and a smaller fraction of beta-strand regions (13.7%). A similar trend was observed in the G glycoprotein-based vaccine construct, with its secondary structure dominated by helical elements, followed by coils and strands. These findings are consistent with another study that reported a comparable pattern of secondary structures (45). The G and F glycoproteins play critical roles in the early stages of hRSV infection (6). Historically, determining whether the G protein was of viral or host origin posed challenges due to variations in the cell lines, virus strains, and protein detection technologies, all of which influenced the observed size and presence of the G protein. Notably, inhibiting the cleavage of the G-protein and incorporating it into a live attenuated RSV vaccine candidate could result in a virus with an intact G protein, leading to a 5-fold increase in infectivity for the nasal epithelium – the primary site of vaccine administration (50).

In combination with the secondary structure data, the three-dimensional modeling using the advanced AI-ML-based Alphafold3 method revealed that the vaccine candidates form well-folded proteins with optimal plDDT values. Molecular docking and dynamics refinement demonstrated that all vaccine candidates exhibit strong interactions with the TLR domain through charged and hydrophobic interactions, forming a tight bonds. The observed interaction patterns align with previously studied vaccine-TLR receptor complex models (51).

The F protein exhibits a higher degree of conservation compared to the G protein, making it the primary target for RSV development. The pre-fusion F protein is the main target of antibody neutralization in the sera of individuals who have experienced multiple RSV infections throughout their lifetime (52). Due to its capacity to elicit a higher concentration of neutralizing antibodies, most vaccine research has focused on the F protein. Prior infection and elevated levels of neutralizing antibodies, particularly those passed down from the mother, provide partial protection against the disease. Moreover, the use of a neutralized F protein mAbs in immunological prophylaxis underscores the critical role of the F protein in RSV vaccine development (53).

In this study, we strategically chose Toll-like receptors (TLRs) 2 and 4 due to their pivotal roles in immune recognition and their established potential as adjuvants, supported by robust literature. TLR2 and TLR4 are integral to the innate immune system, recognizing a diverse array of pathogen-associated molecular patterns (PAMPs) and playing a critical role in the initiation of immune responses (54). This foundational function is essential for the effective development of vaccines, as their activation can significantly enhance the adaptive immune response. Furthermore, existing research underscores the efficacy of targeting these receptors in various vaccine strategies, affirming their relevance and effectiveness in enhancing vaccine efficacy (55). This comprehensive rationale underscores our decision to focus on TLR2 and TLR4, making these vaccine candidates achieve improved immune responses against hRSV.

## 5 Conclusions

This study highlights the promising potential of multi-epitope vaccines developed through immunoinformatics for combating hRSV. We have designed an F and G proteins-based synthetic vaccine that aims to elicit robust immune responses while minimizing adverse effects. The physicochemical characterization of the vaccine constructs indicates favorable properties, including stability and non-allergenic profiles, enhancing their suitability for further development. Additionally, it was demonstrated to stimulate immune responses in both cells and antibodies without triggering type 2 immunity, which are typically associated with RSV infection. This study highlights the potential of bioinformatics-based methods in developing effective therapies for emerging viruses, particularly under constraints such as restricted time and resources. However, these findings are derived from in silico computational analysis and must be validated through experimental studies with *in vivo* and *in vitro* models in laboratory settings. Overall, this research contributes to the ongoing efforts in vaccine innovation, paving the way for effective and safe immunization strategies against hRSV.

## Data Availability

The original contributions presented in the study are included in the article/**Supplementary Material**. Further inquiries can be directed to the corresponding author.
