# Supplementary material for: Design of a peptide-based vaccine against human respiratory syncytial virus using a reverse vaccinology approach: evaluation of immunogenicity, antigenicity, allergenicity, and toxicity
Source: Front Immunol. 2025 Mar 28;16:1546254. doi: 10.3389/fimmu.2025.1546254 (PMC11986473; doi:10.3389/fimmu.2025.1546254)
Supplement: Supplementary file 1 [file DataSheet1.docx]

**Table 1:** Information about targeted proteins retrieved from UniProt Database.

| **Virus** | **Protein** | **Gene** | **Length** | **Mass (Da)** | **Molecule Type** | **Primary Accession ID** |
| --- | --- | --- | --- | --- | --- | --- |
| hRSV | Fusion Glycoprotein | F | 574 AA | 63,751 | Viral cRNA | A0A292GCX4 |
|  | Attachment Glycoprotein | G | 321AA | 35,191 | Viral cRNA | A0A0R9DMI3 |

**Table 2:** An initial assessment of physiochemical properties of F and G glycoproteins using the Expasy protparam tool.

| **Physicochemical Properties** | **Fusion Glycoprotein** | **Attachment Glycoprotein** |
| --- | --- | --- |
| Number of Amino Acids | 574 | 321 |
| Molecular Weight | 63750.57 | 35190.86 |
| Theoretical pI | 9.13 | 9.77 |
| GRAVY (Hydropathicity) | -0.038 | -0.636 |
| Aliphatic index | 102.18 | 68.38 |
| Instability index | 41.81 (unstable) | 35.70 (stable) |
| Extinction Coefficient  (M-1 cm-1, at 280 nm) | 50155 | 20190 |
| Estimated half life | 30 hours (in vitro)  > 20 hours (in vivo)  >10 hours (in vivo) | 30 hours  > 20 hours (in vivo)  >10 hours (in vivo) |

**Table 3.** The physicochemical properties of the final vaccine constructed with F and G proteins.

| **Properties** | **Fusion Glycoprotein** | **Attachment Glycoprotein** |
| --- | --- | --- |
| Number of Amino Acids | 315 | 317 |
| Molecular Weight | 31982.84 | 33292.41 |
| Theoretical pI | 9.46 | 10.07 |
| GRAVY (Hydropathicity) | 0.448 | 0.598 |
| Aliphatic index | 101.40 | 117.22 |
| Instability index | 24.42 (stable) | 20.22 (stable) |
| Extinction Coefficient (M-1 cm-1, at 280 nm) | 30955 | 20860 |
| Estimated half-life | 5.5 hrs (in vitro)  3 mins (yeast, in vivo)  2 mins (*E. coli*, in vivo) | 5.5 hrs (in vitro)  3 mins (yeast, in vivo)  2 mins (*E. coli*, in vivo) |
| Antigenicity | 0.5996 | 0.6048 |
| Allergenicity | Non-Allergenic | Non- Allergenic |
| Toxicity | Non-toxic | Non-toxic |

**B-cell epitopes**

**
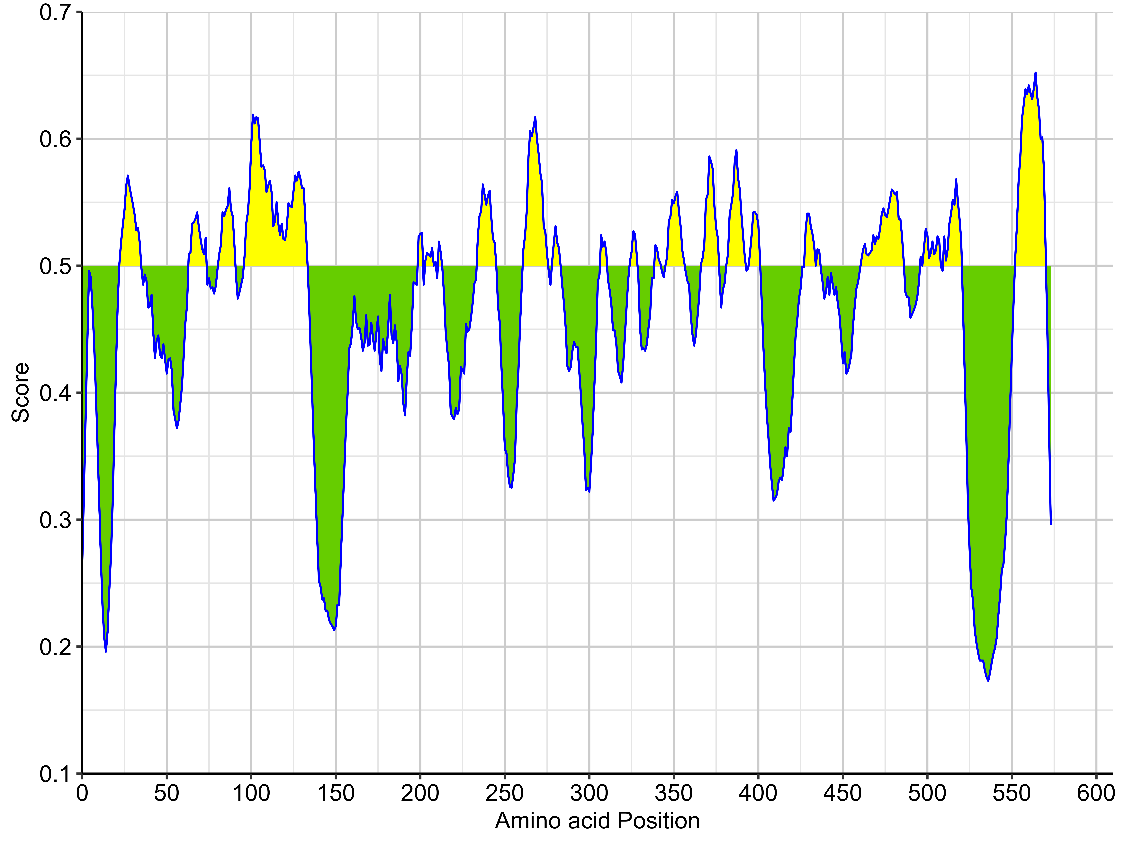
**

**SF1:** Predicted B-cell response inducing F protein epitopes. The green color shows peptides below a threshold score of 0.5 and the yellow color is for peptides above the 0.5 threshold. The color intensity in the IEDB graphical output directly correlated with the suitability of the predicted peptides as potential epitopes, with the brightest yellow regions indicating the most promising candidates.


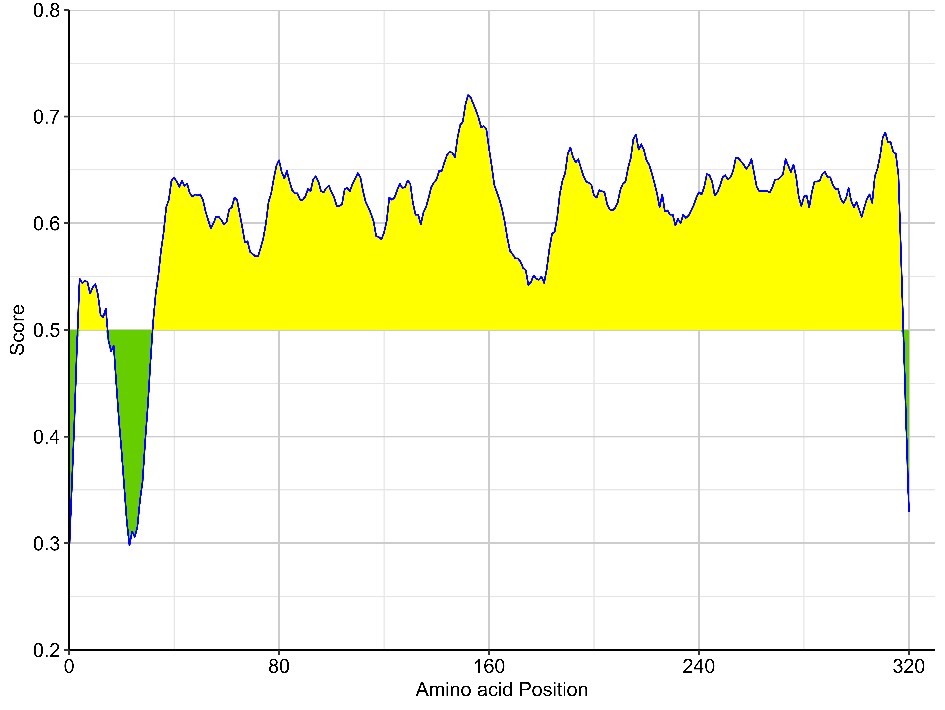


**SF2:** Predicted B-cell response inducing G protein epitopes. The green color shows peptides below a threshold score of 0.5 and the yellow color is for peptides above the 0.5 threshold. The color intensity in the IEDB graphical output directly correlated with the suitability of the predicted peptides as potential epitopes, with the brightest yellow regions indicating the most promising candidates.

**Secondary structures**


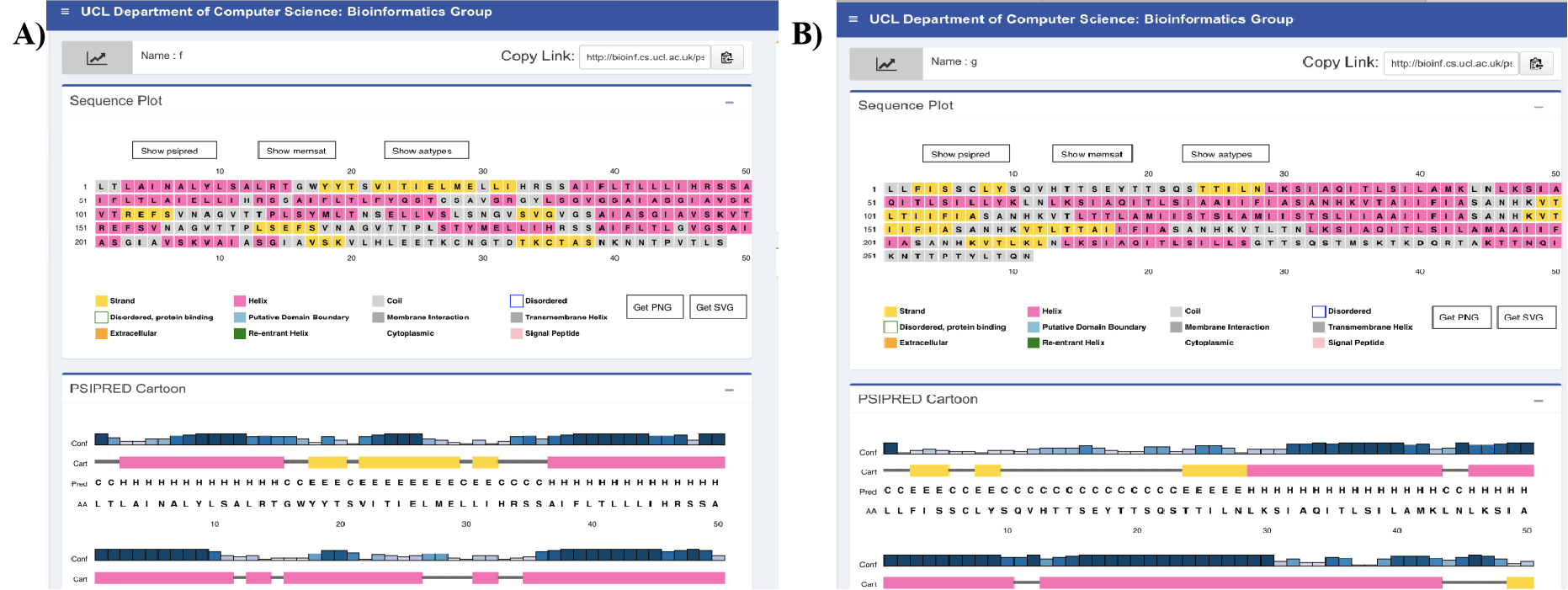


**SF3: S**econdary structure of F glycoproteins predicted by PSIPRED software. Pink color shows helix, yellow shows strands, and grey shows coils.


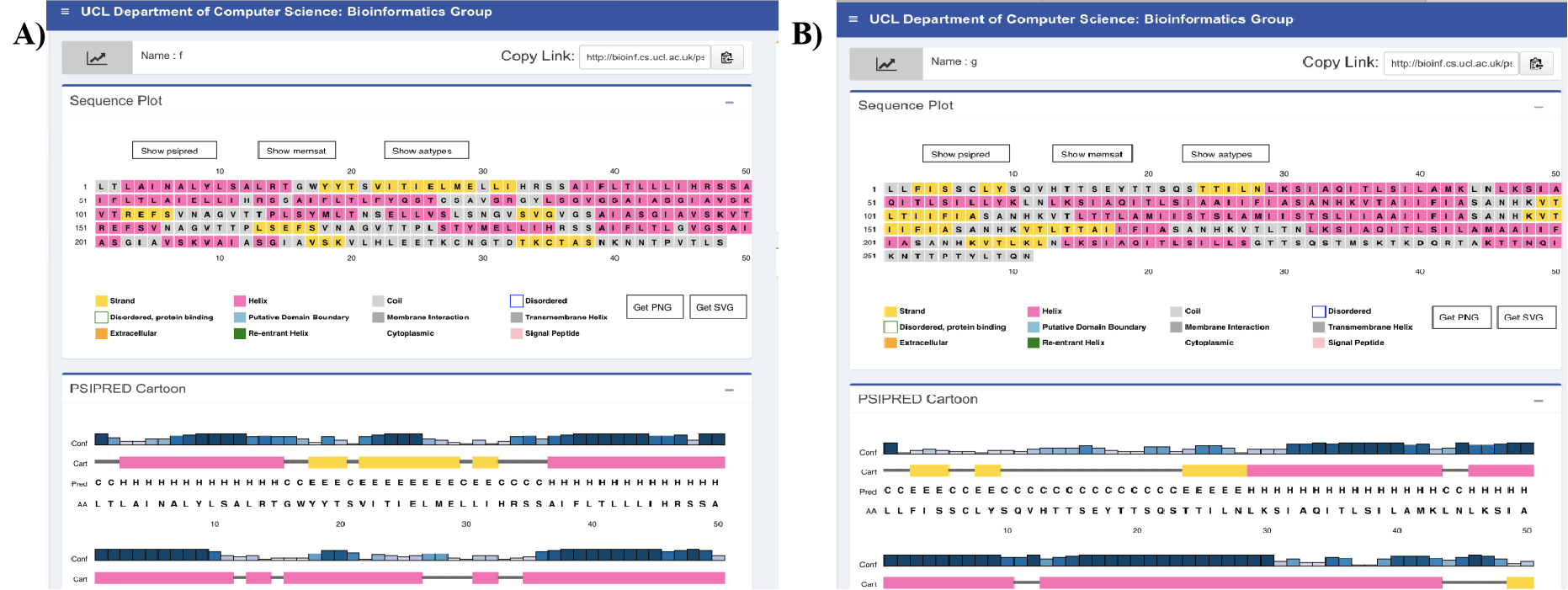


**SF4: S**econdary structure of G glycoproteins predicted by PSIPRED software. Pink color shows helix, yellow shows strands, and grey shows coils.
